# Supplementary material for: A Stomatal Model of Anatomical Tradeoffs Between Gas Exchange and Pathogen Colonization
Source: Front Plant Sci. 2020 Oct 29;11:518991. doi: 10.3389/fpls.2020.518991 (PMC7658178; doi:10.3389/fpls.2020.518991)
Supplement: Appendix 1 — Supplemental methods and spatially implicit model. [file Data_Sheet_1.pdf]

Appendix 1: Supplemental Methods and Spatially Implicit Model

Christopher D. Muir [1*]

[1] *School of Life Sciences, University of Hawaii, Honolulu, Hawaii, USA*

Correspondence*:
Christopher D. Muir
cdmuir@hawaii.edu

SUPPLEMENTAL METHODS

$g_{\text{s,max}}$ calculation

I calculated $g_{\text{s,max}}$ (Equation 1) to water vapor at a reference leaf temperature ($T_{\text{leaf}} = 25°$ C) following Sack and Buckley (2016). They defined a biophysical and morphological constant as:

$$b = D_{\text{wv}}/v$$
$$m = \frac{\pi c^2}{j^{0.5}(4hj + \pi c)}$$

b is the diffusion coefficient of water vapor in air (D_{wv}) divided by the kinematic viscosity of dry air (v). $D_{\text{wv}} = 2.49 \times 10^{-5}$ m^2 s^{-1} and $v = 2.24 \times 10^{-2}$ m^3 mol^{-1} at 25° (Monteith and Unsworth, 2013). For kidney-shaped guard cells, $c = h = j = 0.5$.

f_{S} is proportional to the stomatal pore area index

The stomatal pore area index (SPI; Sack et al., 2003) is calculated as the product of the stomatal density and guard cell length (GCL) squared:

$$\text{SPI} = D \times \text{GCL}^2$$

Assuming that the stomatal radius R is half the GCL, then stomatal size S is equivalent to:

$$S = \pi R^2 = \frac{\pi \times \text{GCL}^2}{4}$$

Based on equation 2, it follows that f_{S} and SPI are proportional:

$$f_{\text{S}} = \frac{\pi \times \text{SPI}}{4}$$

SPATIALLY IMPLICIT MODEL

A limitation of the spatially explicit model is that a pathogen could only infect stomata in the focal triangle where it landed. Here I analyze an alternative, spatially implicit, model that relaxes this assumptions. Instead, I assume that a pathogen can potentially infect any stomate on the leaf. It searches through a random walk and has a continuous, constant probability of encountering a stomate that is determined by stomatal cover (f_S). If $fs << 1$, this can be modeled as a homogeneous Poisson process and the distance x a pathogen must travel before reaching a stomate follows an exponential distribution:

$$f(x) = f_\mathrm{S} e^{-f_\mathrm{S} x}$$

Given the constant death rate per unit distance H, the probability of surviving to distance x is e^{-Hx}. The probability of locating a stomate the probability of surviving to distance x multiplied by $f(x)$ and integrated over all x from 0 to ∞:

$$\begin{aligned} p_\text{locate} &= \int_0^\infty f(x) e^{-Hx} dx \\ &= \int_0^\infty f_\mathrm{S} e^{-(f_\mathrm{S}+H)x} dx \\ &= \frac{f_\mathrm{S}}{H + f_\mathrm{S}} \end{aligned}$$

Substituting p_locate above into Equation 5:

$$\begin{aligned} p_\text{colonize} &= f_\mathrm{S} + (1 - f_\mathrm{S}) p_\text{locate} \\ &= f_\mathrm{S} + (1 - f_\mathrm{S}) \frac{f_\mathrm{S}}{H + f_\mathrm{S}} \\ &= f_\mathrm{S} (1 + \frac{1 - f_\mathrm{S}}{H + f_\mathrm{S}}) \end{aligned}$$

With the spatially implicit model, because pathogens can potentially reach any stomate on the leaf, p_colonize is greater than that in the spatially explicit model for the same value of H. For example, if the pathogen can search forever ($H = 0$), then it will always colonize ($p_\text{colonize} = 1$; Figure S3a). But even when $H > 0$, p_colonize is significantly higher than in the spatially implicit than spatially explicit model for the same f_S because pathogens can potentially colonize any stomate on the leaf.

Whereas the spatially explicit model probably underestimates p_locate for pathogens that can search over long distances, the implicit model overestimates because it assumes that the probability of encountering a stomate is constant (i.e. homogeneous Poisson process). This is not true because stomata are discrete areas on the leaf. If a pathogen is searching far away from a stomate, its probability of encountering a stomate in the near future is lower than that for a pathogen searching near a stomate. This should be modeled as a *nonhomogenous* Poisson process. Future work should derive p_locate for the stomatal anatomies presented here under a nonhomogeneous process.

Despite the quantitative differences in the the spatially explicit and implicit models, they have similar qualitative properties when $H > 0$, which is reasonable since the leaf surface is a relatively hostile environment for most pathogens (see [Introduction]). In both models, p_{colonize} increases with, but is higher than f_{S}. In the spatially explicit model, size-density scaling that preserves p_{colonize} is 1 when $H = 0$ and slightly less than 1 otherwise (Figure 4). In the spatially implicit model, the scaling coefficient is always 1. Rearranging the equation for p_{colonize} above and substituting $f_{\text{S}} = DS$, the following relationship holds:

$$SD = \frac{p_{\text{colonize}} H}{1 - p_{\text{colonize}} + H}$$

Since H is a constant the right-hand side of the equation above is constant for a given value of p_{colonize}. Hence the β_p that would preserve the relationship above is simply 1 (Figure S4).

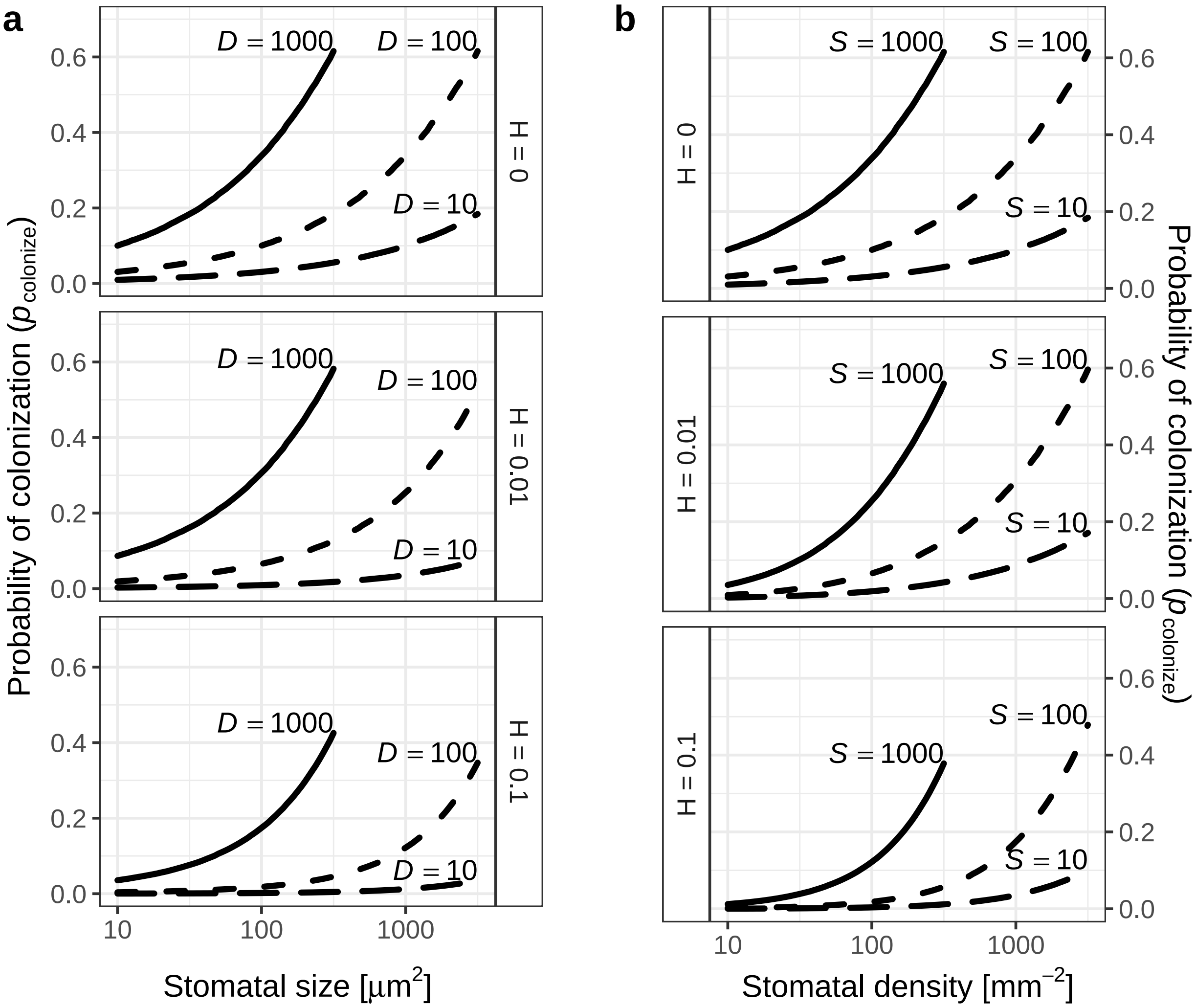

Figure S2. The probability of colonization increases with both stomatal size (S) and density D I simulated the probability of colonization (p_{colonize}, y-axis) over a range of S, D, and H (see [Materials and Methods]) **a.** Each line shows how p_{colonize} increases with S (x-axis, log-scale) for selected values of $D \in \{10, 100, 1000\}$ mm^{-2}. **b.** Each line shows how p_{colonize} increases with D (x-axis, log-scale) for selected values of $S \in \{10, 100, 1000\}$ μm^2. The facets show results for different values of H

Figures

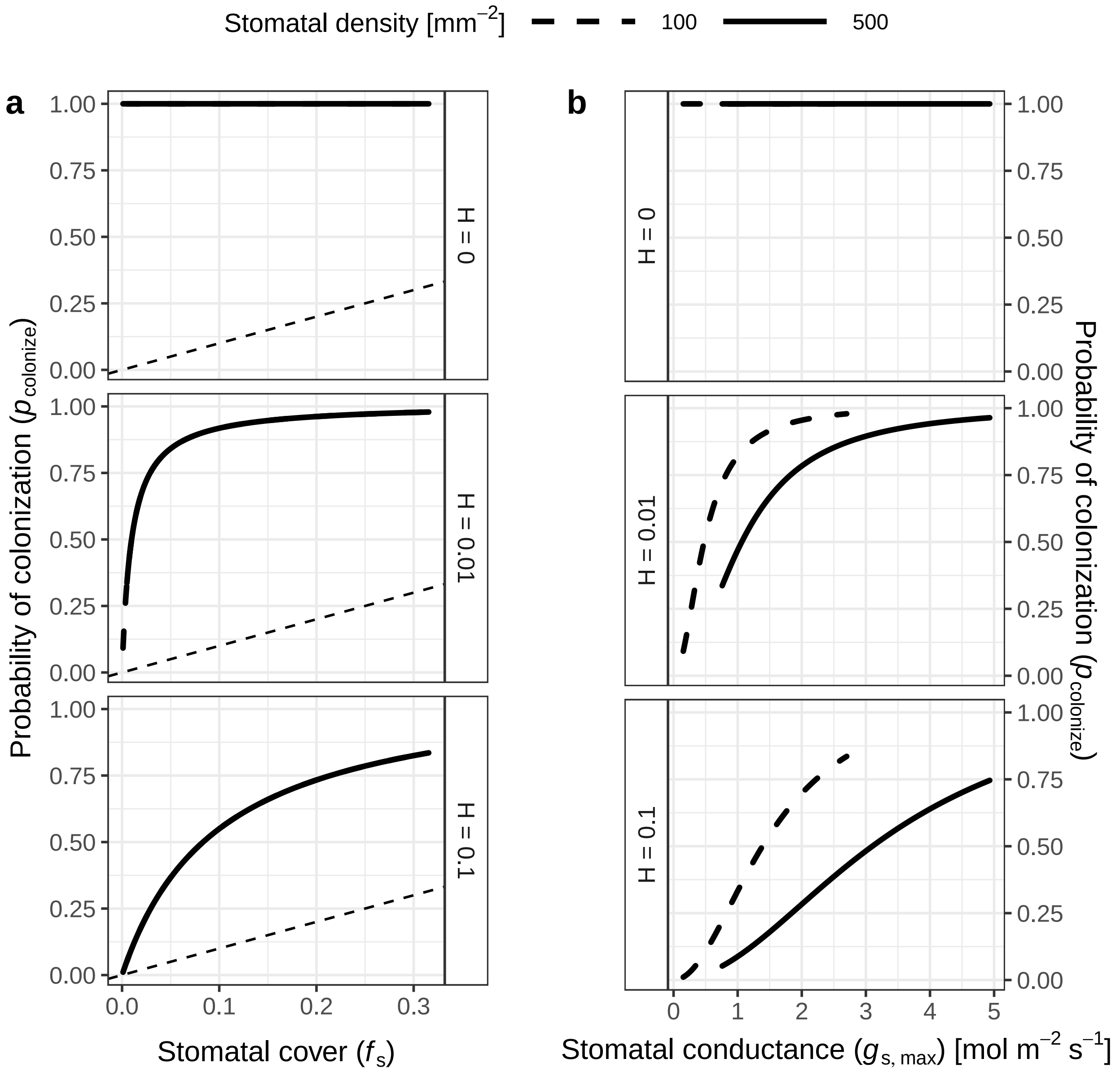

Figure S3. The probability of colonization increases with both stomatal cover and conductance in the spatially implicit model. As in Figure 3, I simulated the probability of colonization (p_{colonize}, y-axis) over a range of stomatal densities and sizes (see [Materials and Methods]), but a subset of results are shown here. Stomatal size and density determine stomatal cover (f_S; Equation 2) and theoretical maximum stomatal conductance ($g_{\mathrm{s,max}}$; Equation 1). **a.** p_{colonize} initially increases rapidly with f_S (x-axis), then slows down to a linear relationship. Overall, p_{colonize} is lower when pathogens can die on the leaf surface ($H > 0$). The relationship between f_S and p_{colonize} is the same regardless of stomatal density for all values of H, which is why the lines overlap. **b.** p_{colonize} increases sigmoidally with $g_{\mathrm{s,max}}$ at all stomatal densities, but p_{colonize} is lower at higher densities for a given $g_{\mathrm{s,max}}$. The relationship between $g_{\mathrm{s,max}}$ and p_{colonize} is similar for all values of $H > 0$.

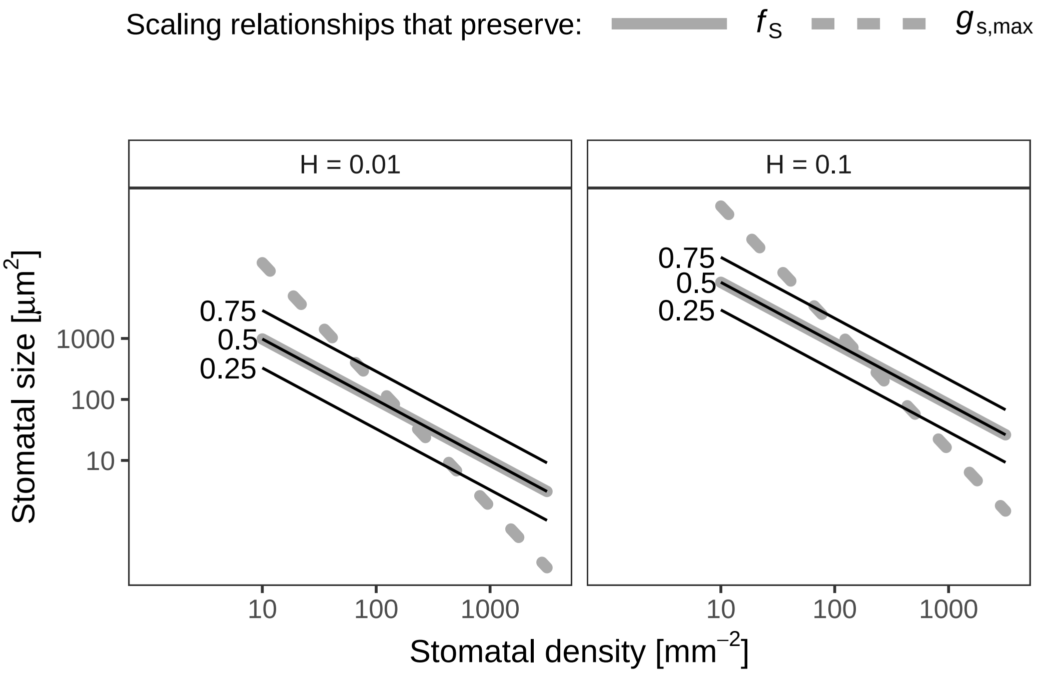

Figure S4. Log-log scaling relationships between stomatal density (D, *x*-axis) and size (S, *y*-axis) that preserve the probability of colonization (p_{colonize}) in the spatially implicit model. As in Figure 4, in each panel, solid lines indicate values of D and S where p_{colonize} is 0.25 (lowest line), 0.5, or 0.75 (highest line). For reference, dashed grey lines show scaling relationships that preserve f_S ($\beta = 1$, slope $= -1/\beta = -1$) and $g_{\text{s,max}}$ ($\beta = 0.5$, slope $= -1/\beta = -2$) drawn through the centroid of the plotting region. The scaling exponent is unity $\beta = 1$ when $H > 0$.

REFERENCES

Monteith, J. L., and Unsworth, M. H. (2013). *Principles of environmental physics: Plants, animals, and the atmosphere*. 4th ed. Amsterdam ; Boston: Elsevier/Academic Press.

Sack, L., and Buckley, T. N. (2016). The developmental basis of stomatal density and flux. *Plant Physiology* 171, 2358–2363. doi:10.1104/pp.16.00476.

Sack, L., Cowan, P. D., Jaikumar, N., and Holbrook, N. M. (2003). The 'hydrology' of leaves: Co-ordination of structure and function in temperate woody species. *Plant, Cell and Environment* 26, 1343–1356. doi:10.1046/j.0016-8025.2003.01058.x.
