# Supplementary material for: A Stomatal Model of Anatomical Tradeoffs Between Gas Exchange and Pathogen Colonization
Source: Front Plant Sci. 2020 Oct 29;11:518991. doi: 10.3389/fpls.2020.518991 (PMC7658178; doi:10.3389/fpls.2020.518991)
Supplement: Appendix 2 — Additional R packages. [file Data_Sheet_2.pdf]

Appendix 2: Additional R packages

Christopher D. Muir [1*]

[1] *School of Life Sciences, University of Hawaii, Honolulu, Hawaii, USA*

Correspondence*:
Christopher D. Muir
cdmuir@hawaii.edu

package	version	reference
assertthat	0.2.1	Wickham (2019a)
bibtex	0.4.2.2	Francois (2020)
BiocManager	1.30.10	Morgan (2019)
cli	2.0.2	Csárdi (2020)
codetools	0.2-16	Tierney (2018)
colorspace	1.4-1	Zeileis et al. (2019)
		Zeileis et al. (2009)
		Stauffer et al. (2009)
cowplot	1.0.0	Wilke (2019)
crayon	1.3.4	Csárdi (2017)
digest	0.6.25	Antoine Lucas et al. (2020)
dplyr	1.0.0	Wickham et al. (2020a)
ellipsis	0.3.1	Wickham (2020)
evaluate	0.14	Wickham and Xie (2019)
fansi	0.4.1	Gaslam (2020)
farver	2.0.3	Pedersen et al. (2020)
filehash	2.4-2	Peng (2006)
furrr	0.1.0	Vaughan and Dancho (2018)
future	1.18.0	Bengtsson (2020)
generics	0.0.2	Kuhn et al. (2018)
ggforce	0.3.2	Pedersen (2020)
ggimage	0.2.8	Yu (2020a)
gginnards	0.0.3	Aphalo (2019)
ggplot2	3.3.2	Wickham (2016)
ggplotify	0.0.5	Yu (2020b)
globals	0.12.5	Bengtsson (2019a)
glue	1.4.1	Hester (2020)
gridGraphics	0.5-0	Murrell and Wen (2020)
gtable	0.3.0	Wickham and Pedersen (2019)
hms	0.5.3	Müller (2020)
htmltools	0.5.0	Cheng et al. (2020)
httr	1.4.1	Wickham (2019b)
jsonlite	1.7.0	Ooms (2014)
knitcitations	1.0.10	Boettiger (2019)
knitr	1.29	Xie (2020a)
		Xie (2015)

package	version	reference
		Xie (2014)
lifecycle	0.2.0	Henry (2020)
listenv	0.8.0	Bengtsson (2019b)
lubridate	1.7.9	Grolemund and Wickham (2011)
magick	2.4.0	Ooms (2020)
magrittr	1.5	Bache and Wickham (2014)
MASS	7.3-51.6	Venables and Ripley (2002)
munsell	0.5.0	Wickham (2018)
pillar	1.4.6	Müller and Wickham (2020a)
pkgconfig	2.0.3	Csárdi (2019)
plyr	1.8.6	Wickham (2011)
polyclip	1.10-0	Johnson and Baddeley (2019)
pracma	2.2.9	Borchers (2019)
purrr	0.3.4	Henry and Wickham (2020a)
R6	2.4.1	Chang (2019)
Rcpp	1.0.5	Eddelbuettel and François (2011)
		Eddelbuettel (2013)
		Eddelbuettel and Balamuta (2017)
readr	1.3.1	Wickham et al. (2018)
RefManageR	1.2.12	McLean (2017)
		McLean (2014)
rlang	0.4.7	Henry and Wickham (2020b)
rmarkdown	2.3	Allaire et al. (2020a)
		Xie et al. (2018)
rticles	0.14	Allaire et al. (2020b)
rvcheck	0.1.8	Yu (2020c)
scales	1.1.1	Wickham and Seidel (2020)
sessioninfo	1.1.1	Csárdi et al. (2018)
stringi	1.4.6	Gagolewski (2020)
stringr	1.4.0	Wickham (2019c)
tibble	3.0.3	Müller and Wickham (2020b)
tidyr	1.1.0	Wickham and Henry (2020)
tidyselect	1.1.0	Henry and Wickham (2020c)
tikzDevice	0.12.3.1	Sharpsteen and Bracken (2020)
tweenr	1.0.1	Pedersen (2018)
units	0.6-7	Pebesma et al. (2016)
vctrs	0.3.1	Wickham et al. (2020b)
withr	2.2.0	Hester et al. (2020)
xfun	0.15	Xie (2020b)
xml2	1.3.2	Wickham et al. (2020c)
yaml	2.2.1	Stephens et al. (2020)

REFERENCES

Allaire, J., Xie, Y., McPherson, J., Luraschi, J., Ushey, K., Atkins, A., Wickham, H., Cheng, J., Chang, W., and Iannone, R. (2020a). *Rmarkdown: Dynamic documents for r*. Available at: `https://github.com/rstudio/rmarkdown`.

Allaire, J., Xie, Y., R Foundation, Wickham, H., Journal of Statistical Software, Vaidyanathan, R., Association for Computing Machinery, Boettiger, C., Elsevier, Broman, K., et al. (2020b). *Rticles: Article formats for r markdown*. Available at: `https://CRAN.R-project.org/package=rticles`.

Antoine Lucas, D. E. with contributions by, Tuszynski, J., Bengtsson, H., Urbanek, S., Frasca, M., Lewis, B., Stokely, M., Muehleisen, H., Murdoch, D., Hester, J., et al. (2020). *Digest: Create compact hash digests of r objects*. Available at: `https://CRAN.R-project.org/package=digest`.

Aphalo, P. J. (2019). *Gginnards: Explore the innards of 'ggplot2' objects*. Available at: `https://CRAN.R-project.org/package=gginnards`.

Bache, S. M., and Wickham, H. (2014). *Magrittr: A forward-pipe operator for r*. Available at: `https://CRAN.R-project.org/package=magrittr`.

Bengtsson, H. (2020). *Future: Unified parallel and distributed processing in r for everyone*. Available at: `https://CRAN.R-project.org/package=future`.

Bengtsson, H. (2019a). *Globals: Identify global objects in r expressions*. Available at: `https://CRAN.R-project.org/package=globals`.

Bengtsson, H. (2019b). *Listenv: Environments behaving (almost) as lists*. Available at: `https://CRAN.R-project.org/package=listenv`.

Boettiger, C. (2019). *Knitcitations: Citations for 'knitr' markdown files*. Available at: `https://CRAN.R-project.org/package=knitcitations`.

Borchers, H. W. (2019). *Pracma: Practical numerical math functions*. Available at: `https://CRAN.R-project.org/package=pracma`.

Chang, W. (2019). *R6: Encapsulated classes with reference semantics*. Available at: `https://CRAN.R-project.org/package=R6`.

Cheng, J., Sievert, C., Chang, W., Xie, Y., and Allen, J. (2020). *Htmltools: Tools for html*. Available at: `https://CRAN.R-project.org/package=htmltools`.

Csárdi, G. (2020). *Cli: Helpers for developing command line interfaces*. Available at: `https://CRAN.R-project.org/package=cli`.

Csárdi, G. (2017). *Crayon: Colored terminal output*. Available at: `https://CRAN.R-project.org/package=crayon`.

Csárdi, G. (2019). *Pkgconfig: Private configuration for 'r' packages*. Available at: `https://CRAN.R-project.org/package=pkgconfig`.

Csárdi, G., core, R., Wickham, H., Chang, W., Flight, R. M., Müller, K., and Hester, J. (2018). *Sessioninfo: R session information*. Available at: `https://CRAN.R-project.org/package=sessioninfo`.

Eddelbuettel, D. (2013). *Seamless R and C++ integration with Rcpp*. New York: Springer doi:10.1007/978-1-4614-6868-4.

Eddelbuettel, D., and Balamuta, J. J. (2017). Extending extitR with extitC++: A Brief Introduction to extitRcpp. *PeerJ Preprints* 5, e3188v1. doi:10.7287/peerj.preprints.3188v1.

Eddelbuettel, D., and François, R. (2011). Rcpp: Seamless R and C++ integration. *Journal of Statistical Software* 40, 1–18. doi:10.18637/jss.v040.i08.

Francois, R. (2020). *Bibtex: Bibtex parser*. Available at: https://CRAN.R-project.org/package=bibtex.

Gagolewski, M. (2020). *R package stringi: Character string processing facilities*. Available at: http://www.gagolewski.com/software/stringi/.

Gaslam, B. (2020). *Fansi: ANSI control sequence aware string functions*. Available at: https://CRAN.R-project.org/package=fansi.

Grolemund, G., and Wickham, H. (2011). Dates and times made easy with lubridate. *Journal of Statistical Software* 40, 1–25. Available at: http://www.jstatsoft.org/v40/i03/.

Henry, L. (2020). *Lifecycle: Manage the life cycle of your package functions*. Available at: https://CRAN.R-project.org/package=lifecycle.

Henry, L., and Wickham, H. (2020a). *Purrr: Functional programming tools*. Available at: https://CRAN.R-project.org/package=purrr.

Henry, L., and Wickham, H. (2020b). *Rlang: Functions for base types and core r and 'tidyverse' features*. Available at: https://CRAN.R-project.org/package=rlang.

Henry, L., and Wickham, H. (2020c). *Tidyselect: Select from a set of strings*. Available at: https://CRAN.R-project.org/package=tidyselect.

Hester, J. (2020). *Glue: Interpreted string literals*. Available at: https://CRAN.R-project.org/package=glue.

Hester, J., Müller, K., Ushey, K., Wickham, H., and Chang, W. (2020). *Withr: Run code 'with' temporarily modified global state*. Available at: https://CRAN.R-project.org/package=withr.

Johnson, A., and Baddeley, A. (2019). *Polyclip: Polygon clipping*. Available at: https://CRAN.R-project.org/package=polyclip.

Kuhn, M., Wickham, H., and Vaughan, D. (2018). *Generics: Common s3 generics not provided by base r methods related to model fitting*. Available at: https://CRAN.R-project.org/package=generics.

McLean, M. W. (2017). RefManageR: Import and manage bibtex and biblatex references in r. *The Journal of Open Source Software*. doi:10.21105/joss.00338.

McLean, M. W. (2014). *Straightforward bibliography management in r using the refmanager package*. Available at: https://arxiv.org/abs/1403.2036.

Morgan, M. (2019). *BiocManager: Access the bioconductor project package repository*. Available at: https://CRAN.R-project.org/package=BiocManager.

Murrell, P., and Wen, Z. (2020). *GridGraphics: Redraw base graphics using 'grid' graphics*. Available at: https://CRAN.R-project.org/package=gridGraphics.

Müller, K. (2020). *Hms: Pretty time of day*. Available at: `https://CRAN.R-project.org/package=hms`.

Müller, K., and Wickham, H. (2020a). *Pillar: Coloured formatting for columns*. Available at: `https://CRAN.R-project.org/package=pillar`.

Müller, K., and Wickham, H. (2020b). *Tibble: Simple data frames*. Available at: `https://CRAN.R-project.org/package=tibble`.

Ooms, J. (2020). *Magick: Advanced graphics and image-processing in r*. Available at: `https://CRAN.R-project.org/package=magick`.

Ooms, J. (2014). The jsonlite package: A practical and consistent mapping between json data and r objects. *arXiv:1403.2805 [stat.CO]*. Available at: `https://arxiv.org/abs/1403.2805`.

Pebesma, E., Mailund, T., and Hiebert, J. (2016). Measurement units in R. *R Journal* 8, 486–494. doi:10.32614/RJ-2016-061.

Pedersen, T. L. (2020). *Ggforce: Accelerating 'ggplot2'*. Available at: `https://CRAN.R-project.org/package=ggforce`.

Pedersen, T. L. (2018). *Tweenr: Interpolate data for smooth animations*. Available at: `https://CRAN.R-project.org/package=tweenr`.

Pedersen, T. L., Nicolae, B., and François, R. (2020). *Farver: High performance colour space manipulation*. Available at: `https://CRAN.R-project.org/package=farver`.

Peng, R. D. (2006). Interacting with data using the filehash package. *R News* 6, 19–24. Available at: `https://cran.r-project.org/doc/Rnews/`.

Sharpsteen, C., and Bracken, C. (2020). *TikzDevice: R graphics output in latex format*. Available at: `https://CRAN.R-project.org/package=tikzDevice`.

Stauffer, R., Mayr, G. J., Dabernig, M., and Zeileis, A. (2009). Somewhere over the rainbow: How to make effective use of colors in meteorological visualizations. *Bulletin of the American Meteorological Society* 96, 203–216. doi:10.1175/BAMS-D-13-00155.1.

Stephens, J., Simonov, K., Xie, Y., Dong, Z., Wickham, H., Horner, J., reikoch, Beasley, W., O'Connor, B., and Warnes, G. R. (2020). *Yaml: Methods to convert r data to yaml and back*. Available at: `https://CRAN.R-project.org/package=yaml`.

Tierney, L. (2018). *Codetools: Code analysis tools for r*. Available at: `https://CRAN.R-project.org/package=codetools`.

Vaughan, D., and Dancho, M. (2018). *Furrr: Apply mapping functions in parallel using futures*. Available at: `https://CRAN.R-project.org/package=furrr`.

Venables, W. N., and Ripley, B. D. (2002). *Modern applied statistics with s*. Fourth. New York: Springer Available at: `http://www.stats.ox.ac.uk/pub/MASS4`.

Wickham, C. (2018). *Munsell: Utilities for using munsell colours*. Available at: `https://CRAN.R-project.org/package=munsell`.

Wickham, H. (2019a). *Assertthat: Easy pre and post assertions*. Available at: `https://CRAN.R-project.org/package=assertthat`.

Wickham, H. (2020). *Ellipsis: Tools for working with ...* Available at: https://CRAN.R-project.org/package=ellipsis.

Wickham, H. (2016). *Ggplot2: Elegant graphics for data analysis*. Springer-Verlag New York Available at: https://ggplot2.tidyverse.org.

Wickham, H. (2019b). *Httr: Tools for working with urls and http*. Available at: https://CRAN.R-project.org/package=httr.

Wickham, H. (2019c). *Stringr: Simple, consistent wrappers for common string operations*. Available at: https://CRAN.R-project.org/package=stringr.

Wickham, H. (2011). The split-apply-combine strategy for data analysis. *Journal of Statistical Software* 40, 1–29. Available at: http://www.jstatsoft.org/v40/i01/.

Wickham, H., François, R., Henry, L., and Müller, K. (2020a). *Dplyr: A grammar of data manipulation*. Available at: https://CRAN.R-project.org/package=dplyr.

Wickham, H., and Henry, L. (2020). *Tidyr: Tidy messy data*. Available at: https://CRAN.R-project.org/package=tidyr.

Wickham, H., Henry, L., and Vaughan, D. (2020b). *Vctrs: Vector helpers*. Available at: https://CRAN.R-project.org/package=vctrs.

Wickham, H., Hester, J., and Francois, R. (2018). *Readr: Read rectangular text data*. Available at: https://CRAN.R-project.org/package=readr.

Wickham, H., Hester, J., and Ooms, J. (2020c). *Xml2: Parse xml*. Available at: https://CRAN.R-project.org/package=xml2.

Wickham, H., and Pedersen, T. L. (2019). *Gtable: Arrange 'grobs' in tables*. Available at: https://CRAN.R-project.org/package=gtable.

Wickham, H., and Seidel, D. (2020). *Scales: Scale functions for visualization*. Available at: https://CRAN.R-project.org/package=scales.

Wickham, H., and Xie, Y. (2019). *Evaluate: Parsing and evaluation tools that provide more details than the default*. Available at: https://CRAN.R-project.org/package=evaluate.

Wilke, C. O. (2019). *Cowplot: Streamlined plot theme and plot annotations for 'ggplot2'*. Available at: https://CRAN.R-project.org/package=cowplot.

Xie, Y. (2015). *Dynamic documents with R and knitr*. 2nd ed. Boca Raton, Florida: Chapman; Hall/CRC Available at: https://yihui.org/knitr/.

Xie, Y. (2014). "Knitr: A comprehensive tool for reproducible research in R," in *Implementing reproducible computational research*, eds. V. Stodden, F. Leisch, and R. D. Peng (Chapman; Hall/CRC). Available at: http://www.crcpress.com/product/isbn/9781466561595.

Xie, Y. (2020a). *Knitr: A general-purpose package for dynamic report generation in r*. Available at: https://yihui.org/knitr/.

Xie, Y. (2020b). *Xfun: Miscellaneous functions by 'yihui xie'*. Available at: https://CRAN.R-project.org/package=xfun.

Xie, Y., Allaire, J. J., and Grolemund, G. (2018). *R markdown: The definitive guide*. Boca Raton, Florida: Chapman; Hall/CRC Available at: `https://bookdown.org/yihui/rmarkdown`.

Yu, G. (2020a). *Ggimage: Use image in 'ggplot2'*. Available at: `https://CRAN.R-project.org/package=ggimage`.

Yu, G. (2020b). *Ggplotify: Convert plot to 'grob' or 'ggplot' object*. Available at: `https://CRAN.R-project.org/package=ggplotify`.

Yu, G. (2020c). *Rvcheck: R/package version check*. Available at: `https://CRAN.R-project.org/package=rvcheck`.

Zeileis, A., Fisher, J. C., Hornik, K., Ihaka, R., McWhite, C. D., Murrell, P., Stauffer, R., and Wilke, C. O. (2019). colorspace: A toolbox for manipulating and assessing colors and palettes. arXiv.org E-Print Archive Available at: `http://arxiv.org/abs/1903.06490`.

Zeileis, A., Hornik, K., and Murrell, P. (2009). Escaping RGBland: Selecting colors for statistical graphics. *Computational Statistics & Data Analysis* 53, 3259–3270. doi:10.1016/j.csda.2008.11.033.
